# Lower-body strength and power profiles and their relationships with shot velocity and accuracy in elite arena soccer players

**DOI:** 10.3389/fspor.2026.1766063

**Published:** 2026-03-17

**Authors:** Minh N. Q. Nguyen, Caleb Bean, Angeleau Scott, Quincy Johnson, Thayne A. Munce, Andrew C. Fry

**Affiliations:** Jayhawk Athletic Performance Laboratory – Wu Tsai Human Performance Alliance, Department of Health, Sport and Exercise Science, University of Kansas, Lawrence, KS, United States

**Keywords:** countermovement jump, elite sports, football, force plate, isometric mid-thigh pull, reactive strength, shooting performance, soccer

## Abstract

**Background:**

Soccer goals depend on the ability to strike the ball both fast and accurately, yet it is unclear which lower-body neuromuscular qualities best underpin these outcomes in professional arena soccer. This study examined how force plate–derived strength and power characteristics relate to shot velocity, accuracy, and a combined shooting proficiency score in elite arena players.

**Methods:**

Thirty-two male Major Arena Soccer League players completed a battery of lower-body tests on dual force plates [isometric mid-thigh pull [IMTP], countermovement jump [CMJ], drop jump, and 10/5 repeated ankle hops], followed 24–48 h later by a standardized Rosch Soccer Shooting Test. Shooting outcomes were dominant-foot shot velocity (VEL; radar-derived), shooting accuracy (ACC; summed target-grid score of the best three shots), and a composite proficiency index (PROF = VEL × ACC). Pearson correlations were used to screen for redundancy among neuromuscular variables, and separate forward-entry multiple regression models (standardized predictors) were run for VEL, ACC, and PROF.

**Results:**

Shot velocity was significantly associated only with CMJ braking impulse (*R* = 0.39, *R*^2^ = 0.15, *p* = 0.03), indicating that players with greater eccentric braking capacity tended to strike the ball faster. Shooting accuracy was best explained by IMTP peak force and 10/5 peak reactive strength index (*R* = 0.60, *R*^2^ = 0.36, *p* < 0.001), while PROF was associated with IMTP relative peak force and 10/5 peak reactive strength index (*R* = 0.55, *R*^2^ = 0.31, *p* = 0.01).

**Discussion:**

In professional arena soccer players, faster shots were linked to superior eccentric braking ability, whereas more accurate and more proficient shooting were associated with greater maximal strength (especially relative to body mass) and ankle-dominant reactive strength. These findings highlight distinct, trainable neuromuscular profiles for powerful vs. precise shooting that can inform targeted strength and plyometric programming in high-performance soccer.

## 1 Introduction

Scoring goals in soccer critically depends on the ability to shoot with both high velocity and accuracy. A faster shot reduces the goalkeeper's reaction time, while accuracy ensures placement beyond the keeper's reach. Elite players consistently outperform sub-elite peers in ball velocity, often reaching average shot speeds of ∼80 km/h compared with ∼74 km/h in university-level players, despite similar accuracy (1, 2). At the youth level, superior dominant-foot shooting velocity and accuracy distinguish national-level from sub-elite players (3). These findings emphasize that velocity is a key separator at higher levels, though accuracy remains equally decisive in match play.

Although the fundamental technical skills of passing and shooting are shared across formats, arena soccer imposes distinct constraints compared with the traditional 11-a-side game. Major Arena Soccer League (MASL) matches, similar to the other small-sized indoor soccer games, are played on an ice-hockey-sized boarded field, with six players per team, unlimited on-the-fly substitutions, and four 15-min quarters, resulting in very frequent high-intensity transitions and a greater density of technical actions and shots per minute than full-size outdoor soccer (4–7). Match- analysis work in indoor and futsal variants shows that payers perform more accelerations, changes of direction, shots in smaller spaces, and spend a large proportion of match time above ∼85%–90% of maximal heart rate, reflecting repeated explosive actions from short run-ups rather than mainly long-distance running (6–8). Consequently, arena players must repeatedly produce powerful, accurate shots under time and space pressure, rendering their lower-body strength, power, and reactive stiffness particularly important for performance in this setting.

Shot velocity is largely determined by foot speed at impact, which depends on neuromuscular force and power (9). Studies across multiple populations consistently report moderate correlations between leg strength or power and ball velocity (*r* ≈ 0.44–0.66) (10–13). Countermovement Jump (CMJ) height, a classic indicator of explosive leg power, shows a strong correlation with dominant-leg ball speed in elite players (*r* ≈ 0.58) (12). Similarly, quadriceps and hamstring isokinetic torque (*r* ≈ 0.56–0.66) (13, 14) and Isometric Mid-Thigh Pull (IMTP) peak force demonstrate significant associations with shot velocity (4). Importantly, intervention studies show that plyometric and explosive training not only increase CMJ height but also improve maximal instep kick velocity by ∼12% (12, 13), highlighting a causal role of explosive neuromuscular qualities in ball striking. However, strength alone does not guarantee faster kicks. For example, youth players with higher combined isometric strength paradoxically showed lower ball speeds (*r* = −0.989), suggesting poor coordination limited force transfer (15). Similarly, soccer players do not always possess superior maximal strength compared with other athletes yet still achieve higher shot speeds (16). This underscores that while strength and power set the physical ceiling for ball velocity, technical skill and coordination determine whether that capacity is effectively utilized (9, 15–17).

Recent biomechanical evidence suggests that the eccentric braking phase of the CMJ may be especially important for powerful kicking (9, 18–22). Rapid braking and force redirection by the support leg are believed to facilitate a more efficient transition into the downswing, potentially making eccentric braking impulse a stronger predictor of shot velocity than jump height itself (9, 21, 22). This possibility provides a theoretical foundation for examining braking-phase variables when evaluating velocity outcomes.

Accuracy depends more on balance, motor control, and coordination. Supporting leg stability and balanced inter-limb strength have been associated with greater precision (4, 23, 24). Conversely, excessive asymmetry or instability may disrupt shot placement. Some evidence also shows that highly reactive, explosive athletes (high RSI) may sacrifice precision, supporting Fitts's Law that faster movements reduce accuracy (25–28). Fitts's speed–accuracy relationship has also been applied to kicking tasks, where faster approaches or rapid swing phases can reduce ball-placement precision unless technical control is sufficient to compensate (21, 27, 29, 30). Yet, at advanced levels, reactive control—rapid stretch–shortening transitions of the support leg—may enhance precision by improving postural stability during fast kicks. In practice, skilled forwards may modulate velocity to maintain accuracy (21, 25, 31), revealing a complex interaction between power and control.

The IMTP is a widely used force plate test capturing maximal and explosive strength through metrics such as peak force, rate of force development, and inter-limb asymmetry (4, 32). IMTP peak force correlates with CMJ jump height, sprint speed, and change-of-direction ability (31). Because the support leg must provide a stable and forceful base during kicking, stronger athletes may better control trunk and lower-limb motion, potentially improving shot accuracy (4, 9, 21–24, 33–35). IMTP metrics show moderate associations with ball velocity (4) and have been linked to shot accuracy through their influence on support-leg stability (23, 24). These measures together allow profiling of both maximal strength and dynamic stability—key physical underpinnings of powerful and accurate shooting.

CMJ force-time analysis provides additional insights into kicking performance. Jump height consistently predicts kick velocity (*r* ≈ 0.44–0.58) (12). Peak power reflects the combined effect of force × velocity, while concentric impulse relates to takeoff velocity and, by analogy, leg-to-ball impulse (12). Eccentric braking RFD and braking impulse highlight the ability to absorb and redirect force, qualities that support rapid plant-and-swing transitions (12, 18, 19). RSI-mod (jump height ÷ time to take-off) integrates explosiveness into a single metric, and higher RSI-mod athletes are likely more elastic-dominant, producing force more rapidly (32, 36). Notably, accuracy does not strongly correlate with CMJ metrics (12), confirming that technical control is the primary determinant of shot placement. However, force-time asymmetry or excessive compliance may indirectly affect precision by destabilizing the plant leg (24, 37, 38).

Leg stiffness and reactive strength support efficient stretch–shortening cycle (SSC) use during the plant and swing phases of kicking (11). Reactive strength indices derived from drop jumps or repeated hops capture the ability to transition rapidly between eccentric and concentric phases (39–42). Optimal stiffness stabilizes the support leg and promotes energy transfer to the ball (37, 38). Biomechanical studies support that greater stiffness of the supporting leg is associated with reduced unwanted trunk rotation and more stable posture during the instep kick (21, 23, 24, 35), which helps maintain the pelvis and trunk oriented toward the intended target.Moreover, as the kicking leg must make rapid, finely controlled ankle stiffness adjustments at ball contact, the short-contact, ankle-dominant reactive rebound ability that assessed through repeated-hop tests, may be particularly relevant for kicking accuracy, because the support leg must execute brief, controlled contacts during the approach and plant phases, and the kicking leg must make rapid, finely controlled ankle adjustments at ball contact (37, 38, 43, 44). Training that enhances these SSC qualities through plyometrics and eccentric loading has been shown to improve explosive performance and kicking-related outcomes in soccer players, supporting the relevance of these neuromuscular characteristics for both kick speed and movement efficiency (17, 45–47).

Despite extensive soccer research, many studies have examined either strength or skill metrics in isolation, rarely integrating comprehensive neuromuscular profiling with standardized shooting assessments (4, 10, 12–14, 25). Furthermore, the distinct demands of arena soccer—characterized by limited space, rapid play, and frequent shooting under pressure—remain largely unexplored. Accordingly, this study investigated professional arena soccer players, integrating force plate-derived metrics (IMTP, CMJ, Drop Jump, and Multi-Rebound tests) with a standardized shooting test of velocity and accuracy. By linking maximal strength, explosive power, stiffness, and elasticity to shooting outcomes, this work aims to identify key neuromuscular determinants of shooting success and provide applied insight for training and performance optimization. We hypothesized that distinct neuromuscular profiles would underpin shot velocity and accuracy, reflecting separate but complementary physical pathways for powerful vs. precise shooting.

## 2 Materials and methods

### 2.1 Study design overview

This study investigated the relationship between lower-body neuromuscular characteristics and soccer shooting performance in professional arena soccer players. A cross-sectional design was employed, in which athletes completed a battery of force-plate assessments to quantify neuromuscular capabilities related to maximal isometric strength, explosive power, and reactive stiffness, followed by a standardized shooting performance test. Pearson correlation coefficients were used to examine relationships between force-plate–derived measures and shooting outcomes. Separate multiple regression analyses were then performed to determine the contribution of key neuromuscular predictors to each dominant-leg shooting outcome (shot velocity, shooting accuracy, and the composite shooting proficiency score).

### 2.2 Subjects

Thirty-two male professional arena soccer players from the Major Arena Soccer League (MASL) volunteered to participate in this study (age = 26.7 ± 4.2 years; height = 1.79 ± 0.06 m; mass = 77.2 ± 8.5 kg). Subjects were included if they (a) were 18 years or older, (b) were currently contracted MASL athletes actively competing during the season, (c) had a minimum of three years of elite-level playing experience, (d) trained ≥5 sessions per week including team practices and strength/conditioning sessions, and (e) were free from lower-limb injuries or medical conditions that could compromise safe performance of maximal strength, jumping, or shooting assessments. Because this study sampled a single professional team, the sample size was defined by the available roster rather than an *a priori* power analysis. All players were familiar with the testing procedures, which were integrated into their in-season training schedule. All testing procedures performed in the present study were approved by the University's Institutional Review Committee (STUDY00149303) and all subjects signed an Informed Consent Form.

### 2.3 Testing procedures

#### 2.3.1 Testing overview

Testing was conducted over a single week during the in-season period. Each session was completed indoors on a textured synthetic turf surface. Day 1 consisted of the countermovement jump (CMJ), drop jump (DJ), repeated ankle hop test (10/5), and isometric mid-thigh pull (IMTP). Day 2 consisted of the Rosch Soccer Shooting Test (RSST). Before data collection on day 1, each subject's age, height, and body mass were recorded. Height was measured barefoot using a wall-mounted stadiometer (SECA, Hamburg, Germany), and body mass was recorded using electronic digital scales (Tanita Corporation, Tokyo, Japan).

All force-plate tests were conducted using a dual-force plate system (Hawkin Dynamics Inc., Westbrook, ME, USA) sampling at 1,000 Hz, which has been validated as a reliable alternative to laboratory-grade plates for isometric strength, vertical jump, and reactive strength measurements (33, 34, 39, 48). Raw force-time data were processed using Hawkin Dynamics software. A standardized warm-up consisting of 10 min of dynamic mobility drills, progressive sprints, and low-intensity jumps was completed prior to each testing session. Subjects were tested at the same time of day for both sessions, did not eat for 3 h before testing, and refrained from intensive exercise or stimulants in the 24 h prior. Following soccer players physical performance assessment guide (49), recovery intervals were standardized, with at least 3 min between IMTP and 60 s between shooting attempts and jump trials.

All strength and jumping assessments used in this study have demonstrated good-to-excellent test–retest reliability in previous research. A systematic review of the isometric mid-thigh pull reported intraclass correlation coefficients (ICCs) ranging from 0.73 to 0.99 (median ICC = 0.96) and coefficients of variation (CVs) from 0.7% to 11.1% for peak force across 16 studies, indicating consistently high reliability for IMTP peak force measures (50). Countermovement jump height measured via force plates has similarly shown very high reliability (ICC ≈ 0.98), and drop-jump and 10/5 repeated jump protocols used to derive reactive strength index and stiffness demonstrate ICCs in the ∼0.87–0.98 range with coefficients of variation generally <10% in athletic populations (5, 40–42).

#### 2.3.2 Isometric mid-thigh pull (IMTP)

During the maximal isometric strength testing session, participants were weighed on the plates in a quiet stance prior to each trial. Athletes were then positioned in a custom IMTP rack with the barbell secured at the mid-thigh level, corresponding to an approximate knee angle of 125–135° and hip angle of 140–150°, consistent with previously described protocols (33, 48, 51). Participants adopted a clean grip with hands placed shoulder-width apart and elbows fully extended, with the torso upright and shoulders positioned directly over the bar. Following two familiarization pulls at ∼50% and ∼75% effort, each athlete performed 3 maximal efforts, with a minimum of 3-min rest between trials. For each trial, participants were instructed to “pull as hard and as fast as possible” against the immovable bar, and to avoid any countermovement prior to contraction. Verbal encouragement was provided for all attempts. Variables extracted included peak force (N), relative peak force (N·kg^−1^), impulse (N·s), time to peak force (ms), and rate of force development (RFD), calculated as the slope of the force-time curve between 100 and 200 ms after force onset.

#### 2.3.3 Countermovement jump (CMJ)

Participants completed a standardized warm-up consisting of submaximal squat jumps and progressive vertical jumps prior to testing. During CMJ testing, participants stood upright on the plates with hands placed firmly on the hips to eliminate the influence of arm swing. From this position, participants were instructed to perform a rapid downward countermovement to a self-selected depth and then immediately jump vertically with maximal effort. Three maximal CMJs were performed with 60-s rest between trials. Force-time variables were extracted in accordance with established CMJ biomechanical guidelines (20, 39), including jump height (cm, flight-time method), concentric peak force (N), concentric peak power (W), concentric impulse (N·s), eccentric peak force (N), eccentric braking impulse (N·s), eccentric rate of deceleration (N·s^−1^), and eccentric–concentric coupling time (ms), RSI-mod for the countermovement jump (jump height in meters divided by time to take-off in seconds). CMJ Stiffness was computed during the braking phase as the ratio of change in vertical ground reaction force to center-of-mass displacement and reported as a positive magnitude in N·m^−1^.

#### 2.3.4 Drop jump (DJ)

Participants stepped forward off a standardized 30-cm box, landed with both feet on the plates, and were instructed to “jump as high and as fast as possible” while minimizing ground contact time. Three maximal efforts were recorded, separated by 60-s of rest. Outcome measures were derived in accordance with established DJ reactive-strength methodology (52) and included jump height (cm), ground contact time (ms), peak vertical force (N), and reactive strength index [DJ RSI; jump height (m) ÷ ground contact time (s)]. DJ Stiffness was computed during the braking phase as the ratio of change in vertical ground reaction force to center-of-mass displacement and reported as a positive magnitude in N·m^−1^.

#### 2.3.5 Repeated ankles hop test (10/5)

Stretch–shortening cycle efficiency was further assessed using a repeated bilateral ankle hop protocol. Participants performed 10 consecutive ankle hops with hands placed on hips, focusing on minimal ground contact time, minimal bending knees and hips, and maximal rebound height. To reduce noise associated with initial and terminal repetitions, the average of the five middle best hops was used for analysis, consistent with established SSC and stiffness-assessment procedures (5, 40–42). Variables extracted included ground contact time (ms), flight time (ms), reactive strength index–modified (RSI-mod), and bilateral leg stiffness (kN·m^−1^), calculated from braking-phase vertical force and center-of-mass displacement.

#### 2.3.6 Shooting performance

The Rosch Soccer Shooting Test (RSST) was conducted to evaluate players' shooting velocity and accuracy under standardized conditions. Players were required to strike stationary balls from 16 m toward a target grid placed in the goalmouth, following the rules and parameters outlined in the original F-MARC protocol (53) and subsequent applications in elite youth players (3, 54). The grid consisted of a 10 × 5 matrix with numerical values assigned to each zone (range: 0–10), such that shots placed closer to the top corners received higher scores while central and off-target attempts received lower scores. This target grid and related shooting protocols have shown satisfactory test–retest reliability and the ability to discriminate between playing standards, supporting both their reliability and construct validity as measures of soccer shooting skill (3, 5, 54–56). Composite indices that combine ball speed and placement—such as multiplying shot velocity by accuracy—have also demonstrated good measurement properties and strong discrimination between competitive levels in recent validation work (2, 3).

Accuracy in the present study was quantified using the grid score, and a composite shooting proficiency score was calculated as the product of accuracy and shot velocity values, consistent with prior approaches to soccer skill assessment (2, 3, 54–57). Each subject attempted six shots with the dominant foot, alternating aim between left and right target. Ball velocity was measured using a radar gun (Stalker ATS II, Applied Concepts Inc., Richardson, TX, USA). Players were familiarized with the RSST protocol by completing at least five practice shots. Only one player was tested at a time to ensure consistency. Additionally, two tripod-mounted iPhone 14 cameras (Apple Inc., Cupertino, CA, USA) were positioned behind and at an oblique angle to the grid for video verification of ball placement and scoring.

Three primary shooting outcomes were derived from the RSST. Shot velocity (km/h) was defined as the ball speed recorded by the radar gun for each attempt. Shooting accuracy (a.u.) was defined as the sum of grid scores from the three highest-scoring shots out of six attempts, chosen to represent each player's best achievable accuracy while reducing the influence of outlier errors. A composite shooting proficiency score (a.u.) was calculated as the product of shot velocity and shooting accuracy (Shot velocity × Shooting accuracy), providing an integrated index of speed–accuracy performance adapted from Engler et al. (2).

### 2.4 Statistical analysis

All data are expressed as mean ± SD, and 95% confidence intervals (CI) were calculated for all primary variables. Descriptive statistics were computed for all shooting performance and neuromuscular metrics. Pearson correlation coefficients were used to examine common variance among variables and identify redundancy among related measures. When moderate-to-high correlations (*r* ≥ 0.60) were observed between metrics, redundant variables were removed for subsequent analyses. This variable-reduction process (conducted within and across tests) reduced the initial pool of force-plate metrics to four non-redundant candidates for regression entry: (1) CMJ breaking impulse, (2) IMTP peak force, (3) IMTP relative force, and (4) multi-hop RSI.

Separate forward-entry multiple regression analyses (*p* ≤ 0.05 for entry) were performed to determine the contribution of selected neuromuscular predictors to each dominant-leg shooting outcome (shot velocity, shot accuracy, and the composite shooting proficiency score). To reduce risk of overfitting, we constrained the number of predictors in multivariable models relative to the sample size and prioritized parsimonious models based on *a priori* hypotheses and established recommendations for regression model complexity. All predictors and outcomes were standardized (*z*-scores) prior to analysis, so that regression coefficients (*β*) represent standardized beta weights. For each model, the multiple correlation coefficient (*R*), coefficient of determination (*R*^2^), adjusted *R*^2^, *F* statistic, and associated *p*-value were used to evaluate model fit. Standardized beta coefficients, standard errors, and t-values were examined to determine the relative influence of each predictor. A *post-hoc* power analysis (G*Power 3.1, Heinrich Heine University, Düsseldorf, Germany) was conducted to demonstrate the sufficiency of the number of variables retained in the final model. The percentage of total variance explained by each predictor was estimated as the product of its standardized beta coefficient and zero-order correlation (*β* × *rγx*) and expressed as both total and proportional explained variance. Variance inflation factors (VIF) were inspected to assess multicollinearity (all VIF < 1.5), and residual plots were visually examined to confirm linearity, homoscedasticity, and normality of residuals. Statistical significance was set at *p* ≤ 0.05. All analyses were performed in R software, version 4.0.1 (R Core Team, Vienna, Austria; https://www.Rproject.org).

## 3 Results

Descriptive characteristics for the players are presented in Table 1. An overview of the force-plate-derived neuromuscular variables considered for regression analyses, grouped by physiological construct, is shown in Table 2. Descriptive statistics for shooting performance and selected neuromuscular variables are provided in Table 3. Mean shot velocity, shooting accuracy, and composite shooting proficiency scores, as well as selected IMTP, CMJ, DJ, and 10/5 test variables, are reported as mean ± SD with corresponding 95% confidence intervals.

**Table 1:** Descriptive characteristics of professional arena soccer players (mean ± SD).

Variable [unit]	M ± SD
Physical characteristics	
Age (yrs)	26.7 ± 4.2
Height (cm)	179.6 ± 6.9
Body mass (kg)	77.2 ± 8.3

Values are presented as Mean ± SD.

**Table 2:** Description for force-plate–derived neuromuscular constructs and candidate metrics.

Construct	Representative variable^a^	Description
Maximal Strength/Support-Leg Stability	IMTP Peak Force* IMTP Rel. Peak Force* IMTP Impulse 0–250 Ms IMTP RFD 0–250 Ms	Ability to generate high isometric force through the lower body, interpreted as the strength capacity that stabilizes the support leg and whole-body posture during the shot (31–33, 45, 48, 51, 58).
Eccentric Braking Capacity	CMJ Eccentric Braking Impulse* CMJ Eccentric Peak Force CMJ Eccentric Rate Of Deceleration CMJ Stiffness	Capacity to decelerate the center of mass rapidly during the downward phase of a countermovement and transition into propulsion (18–20). Conceptually, this maps onto the plant-and-stop action of the support leg in kicking.
Propulsive Explosive Power	CMJ Jump Height CMJ Concentric Impulse CMJ Concentric Peak Force CMJ RSI-Mod CMJ Time To Take-Off	Concentric force and power production that determine vertical jump height and “explosiveness” in slower stretch–shortening actions. These metrics are strongly interrelated and typically load on a common “propulsive power” or “explosive jump performance” factor rather than representing distinct constructs (20, 36).
Ankle-Dominant Reactive Strength	10/5 Peak RSI* 10/5 Average RSI 10/5 Contact Time 10/5 Flight Time 10/5 Stiffness	Efficiency of short-contact stretch–shortening actions, particularly at the ankle–foot complex. Repeated ankle-hop tests provide reliable measures of peak RSI and leg stiffness and are used to profile reactive “spring-mass” behavior of the lower limbs (5, 38, 41, 42, 59, 60). In the context of kicking, this construct reflects a “quiet,” responsive ankle–foot complex accuracy at high speeds.
Loaded Reactive Strength	DJ RSI DJ Jump Height DJ Contact Time DJ Stiffness DJ Time To Peak Braking	Whole-body reactive strength under higher eccentric loading (step-off drop and rebound). Drop-jump metrics are commonly used to assess fast SSC function and plyometric adaptations (20, 37, 52).

CMJ, countermovement maximal jump; DJ, drop jump; RSI, reactive strength index; RFD, rate of force development; IMTP, isometric mid-thigh pull.

a Variables within each construct were conceptually related and often moderately-to-highly correlated; redundancy screening was used to retain a representative metric when correlations were high (see Supplementary Table S1).

* Metrics were retained as predictors in the final multiple regression models; other metrics were excluded during redundancy checks but still inform the construct-level interpretation.

**Table 3:** Descriptive data (M ± SD and 95% confidence intervals) for performance variables.

Variable [unit]	M ± SD	95% CI lower	95% CI upper
Shooting performance			
Accuracy (score)	16.48 ± 8.47	13.43	19.53
Shot velocity (km/h)	92.69 ± 7.42	90.01	95.37
Composite score (vel×acc)	1,512.84 ± 721.76	1,252.62	1,773.06
Isometric mid-thigh pull (IMTP)			
Peak force (N)	2,876.64 ± 360.41	2,746.70	3,006.58
Relative Peak Force (N/Kg)	36.38 ± 3.87	34.98	37.78
RFD 0–250 ms (N/s)	5,138.62 ± 922.65	4,805.97	5,471.27
Countermovement jump			
Jump height (cm)	39.75 ± 7.11	37.19	42.31
Time to takeoff (s)	0.91 ± 0.09	0.88	0.94
Braking impulse (N·s)	216.27 ± 44.05	200.39	232.15
Drop jump (DJ)			
Time to peak braking force (ms)	76.09 ± 58.99	54.82	97.36
Repeated Ankle hops (10/5)			
Peak RSI (ratio)	2.70 ± 0.78	2.42	2.98
Avg. jump height (cm)	12.33 ± 2.29	11.50	13.16

RFD, rate of force development; RSI, reactive strength index.

Values are presented as Mean ± SD with 95% Confidence Intervals (CI).

All data represent dominant foot performance.

The composite proficiency score (velocity × accuracy) is adapted from Engler et al. (2).

**Figure 1:**
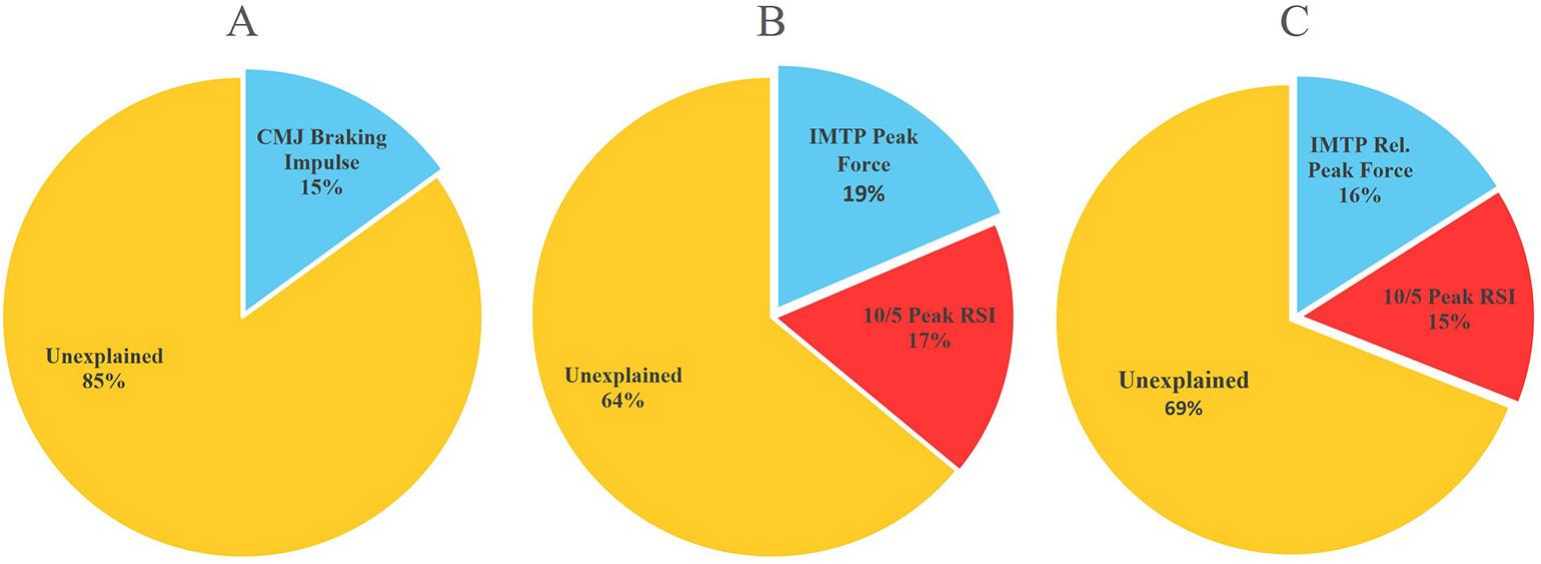
Partitioning of explained and unexplained variance in shooting outcomes from multiple regression models. **(A)** Percentage of total variance in shot velocity accounted for by countermovement jump (CMJ) braking impulse and the remaining unexplained variance. **(B)** Percentage of total variance in shooting accuracy accounted for by isometric mid-thigh pull (IMTP) peak force and 10/5 repeated-hop peak reactive strength index (RSI), with unexplained variance shown. **(C)** Percentage of total variance in the composite shooting proficiency score (velocity × accuracy) accounted for by IMTP relative peak force (body-mass normalized) and 10/5 peak RSI, with the remaining unexplained variance. All percentages are derived from standardized forward-entry multiple regression models (*n* = 32).

Forward-entry multiple regression analyses (*p* ≤ 0.05) were used to examine how force-plate testing metrics were associated with shot velocity, shooting accuracy, and composite shooting proficiency. Results of these analyses are shown in Table 4. Prior to modeling, variables within each construct were screened for redundancy (|*r*| > 0.60), and a reduced set of non-redundant candidate predictors was retained (Supplementary Table S1). For shot velocity, CMJ braking impulse entered at Step 1 and remained the only retained predictor, which significantly explained variance in ball velocity [*R* = 0.39, *R*^2^ = 0.15, adjusted *R*^2^ = 0.12, *F*(1, 30) = 5.34, *p* = 0.03]. For shooting accuracy, IMTP peak force entered at Step 1 [*R*^2^ = 0.17; adjusted *R*^2^ = 0.14; *F*(1,30) = 6.05, *p* = 0.02]. The addition of 10/5 peak RSI at Step 2 significantly improved model fit [Δ*R*^2^ = 0.19, *F*-change(1,29) = 8.80, *p* = 0.006]. The final accuracy model retained IMTP peak force and 10/5 peak RSI [*R* = 0.60; *R*^2^ = 0.36, adjusted *R*^2^ = 0.32; *F*(2,29) = 8.18, *p* < 0.001]. The percentage of total and explained variance for each variable showed that IMTP peak force accounted for 18.6% of the total variance (51.6% of the explained variance), whereas 10/5 peak RSI accounted for 17.4% of the total variance (48.4% of the explained variance). For the composite shooting proficiency score, IMTP relative peak force entered at Step 1 (*R*^2^ = 0.18; adjusted *R*^2^ = 0.15), and adding 10/5 peak RSI at Step 2 significantly increased explained variance [Δ*R*^2^ = 0.13; *F*-change(1,29) = 5.28, *p* = 0.03]. The final proficiency model retained IMTP relative peak force and 10/5 peak RSI [*R* = 0.55; *R*^2^ = 0.31; adjusted *R*^2^ = 0.26; *F*(2,29) = 6.44, *p* = 0.01]. IMTP relative peak force contributed 16.0% of the total variance (52% of the model *R*^2^) and 10/5 peak RSI contributed 15.0% of the total variance (48% of the model *R*^2^). Final model coefficients are provided in Table 4, and the forward-entry sequence is detailed in Supplementary Table S1. Figure 1 summarizes explained vs. unexplained variance. All models met assumptions for linearity and homoscedasticity, variance inflation factors (VIF) were <1.5, and residual diagnostics supported normality of errors. *Post-hoc* power analyses based on observed effect sizes indicated high power for the accuracy and proficiency models (≈0.95 and ≈0.89, respectively) and moderate power for the velocity model (∼0.62), consistent with the smaller proportion of variance explained.

**Table 4:** Regression results for force-plate testing metrics regressed against shooting outcomes.

Outcome	Predictor	*β* (std)	SE	*t*	*p*	% total variance	% explained variance
Shot Velocity	CMJ Braking Impulse	0.39	0.17	2.31	.03	15.0	100.0
Shooting Accuracy	IMTP Peak Force	0.48	0.15	3.18	<.001	18.6	51.6
	10/5 Peak RSI	0.45	0.15	2.96	.01	17.4	48.4
Composite Shooting Proficiency	IMTP Rel PF	0.38	0.16	2.44	.02	16.0	52.0
	10/5 Peak RSI	0.36	0.16	2.29	.03	15.0	48.0

Standardized beta coefficients (*β*), standard errors (SE), *t*-statistics, *p*-values, and the percentage of total and explained variance contributed by each predictor.

## 4 Discussion

### 4.1 Overview of key findings

This study examined the dual demands of kicking power and precision in professional arena soccer, and the results highlight distinct neuromuscular predictors for each outcome. Consistent with evidence that shot velocity is a key differentiator across levels of play (2, 3), our regression models of neuromuscular qualities explained a modest but meaningful portion of variance in standardized shooting performance (∼12%–32%)(Speed Adjusted *R*^2^ ≈ .12; Accuracy Adjusted *R*^2^ ≈ .32; Composite Adjusted *R*^2^ ≈ .26; *n* = 32). In this cohort, shot velocity and accuracy were associated with different physical qualities: faster shots were uniquely related to greater eccentric braking impulse during a countermovement jump (CMJ), whereas shooting accuracy (and a combined speed–accuracy score) was related to a combination of maximal strength (isometric mid-thigh pull force) and reactive strength (repeated-hop reactive strength index).

Practically, these findings indicate that, for these professional arena soccer players, a homogeneous professional sample, superior shot velocity tended to coincide with better eccentric braking ability, whereas higher accuracy and proficiency scores tended to coincide with greater maximal strength and more efficient stretch–shortening cycle function. These results reinforce the importance of both power and control in soccer shooting (2, 3, 53, 57) while providing more detail on which neuromuscular attributes were most strongly associated with each aspect of shooting performance. In a highly skilled task such as shooting—where technical execution, perception–action coupling, and context-specific decision-making are major determinants—these magnitudes are expected, particularly in a homogeneous professional sample where between-player variance in physical capacity is narrower than in mixed-level or developmental cohorts. In applied elite sport performance settings, even a ∼10%–30% signal linked to trainable neuromuscular qualities may be meaningful.

Although only a subset of variables entered the final models, several other force-plate metrics were highly correlated with the retained predictors [e.g., multiple 10/5 RSI metrics and CMJ variables showed strong inter-correlations (*r* ≈ .72–.88) with the predictors that entered the models]. This pattern suggests that many related variables likely reflect the same underlying neuromuscular qualities, and their exclusion should be interpreted as statistical redundancy rather than a lack of relevance. Our variable-reduction approach was intended to prevent redundant measures from inflating model fit and to improve interpretability for practitioners.

While significant, the models explained 12%–32%of the variance across outcomes, meaning that 68%–88% of variance remained unexplained by these force-plate metrics alone. This is expected in a high-skill task in which soccer shooting success depends heavily on technical execution and decision-making (9). Practically, our results indicate that a meaningful, potentially trainable portion of shooting performance is linked to lower-body strength, jump, and reactive rebound qualities, while the majority reflects sport-specific skill execution.

### 4.2 Force-plate metrics and shooting velocity

Focusing first on shot velocity, our results are consistent with and add nuance to prior work linking lower-body power to velocity. Previous studies have reported moderate correlations between vertical jump performance and instep kick velocity (*r* ∼ 0.4–0.6) (12), as well as positive effects of strength/power training on shot velocity in soccer (16, 17). We found that the eccentric braking impulse in the CMJ—rather than jump height or general strength—was the only neuromuscular variable that remained in the model for shot velocity, accounting for about 12% of its variance (adjusted *R*^2^ ≈ 0.12). In other words, players who could generate a larger braking force and impulse when rapidly decelerating and reversing direction (as in the downward phase of a jump) tended to shoot the ball faster. Mechanistically, this makes sense: a firm, rapid “brake” with the supporting leg allows for a quicker transfer from approach to swing, enabling higher swinging leg's foot velocities at foot-ball impact (9). Biomechanical analyses of kicking have likewise emphasized that a strong vertical braking force in the supporting leg (while controlling knee/hip flexion) leads to greater shot velocity (9, 21). In kicking, the braking impulse of the supporting leg is more crucial as the body must decelerate forward momentum so that the swinging limb can accelerate unimpeded (9, 21). Our data are consistent with this principle: players with superior ability to absorb and effectively redirect force (i.e., high CMJ braking impulse) achieved higher shot velocities, whereas measures of maximal strength (IMTP peak force) did not enter the speed model once braking was considered.

This finding helps refine the general notion that “stronger = harder shot” by highlighting rapid eccentric force application as one neuromuscular characteristic associated with higher shot velocity in this cohort. It also helps explain the mixed results in past studies that used jump height alone as a power index for kicking (9, 11, 12, 18, 19, 38) since jump height is an outcome influenced by many factors, but our results indicate the eccentric-phase performance (braking impulse) is an important contributor to generating shot velocity more so than jump height itself.

Practically, coaches might emphasize drills that teach quick, stiff plant and recoil (e.g., short ground-contact plyometrics) to improve a player's ability to generate this braking force, especially with the supporting leg, thereby boosting shot velocity. Moreover, the lack of additional benefit from IMTP maximal strength in the velocity model—and similar observations that isometric or early-phase force measures show no clear correlation with shot velocity (31)—might suggest that once a basic level of strength is achieved by professional players, further gains in absolute strength may not translate into faster shots unless accompanied by improvements in how that strength is applied in the dynamic context of a kick. In this sample, IMTP RFD metrics did not add unique variance beyond peak force, suggesting collinearity with maximal strength rather than a distinct contribution to these shooting outcomes. In summary, achieving maximum shot velocity appears to depend on how force is used—a hard and rapid braking action—rather than simply how much force an athlete can produce.

### 4.3 Force-plate metrics and shooting accuracy and proficiency

In contrast, the determinants of shooting accuracy and proficiency showed a different neuromuscular profile centered on strength and reactive control. Our models indicated that players with higher maximal isometric force (IMTP peak force—for absolute accuracy; and IMTP force relative to body mass—for the proficiency score) and better reactive strength index on a repeated hopping test tended to shoot more accurately. These findings align with prior reports linking lower-limb strength, balance, and stability to kicking precision (24, 35). For example, better single-leg balance (a proxy for support-leg stability) has been associated with improved aim in kicking (35), and insufficient strength in the support leg can impair accuracy (24). Maximal strength capacity likely contributes to accuracy by providing a more stable base and finer control during the plant and swing phases of the shot. In Australian Rules football—a sport also requiring accurate long kicks—athletes with greater relative leg strength and more balance in term of strength between limbs were significantly more accurate on goal (24).

Our results echo these observations: the inclusion of IMTP peak force in the accuracy model and IMTP relative force in the proficiency model suggests that stronger players (especially relative to their body weight) can better stabilize and control the shot, leading to more consistent ball placement with sufficient shot velocity. Reactive strength (as measured by the 10/5 hop RSI) further distinguished the more accurate shooters. This metric reflects the efficiency of the stretch–shortening cycle during quick, small-amplitude hops—in essence, how quickly and elastically an athlete can transition from loading to unloading. A higher RSI in the repeated-hop test implies the ability to produce force with minimal ground contact time, which in the context of a soccer shot likely translates to a “quiet,” fast plant of the supporting leg that minimizes disruption to the kicking motion.

A higher RSI in the repeated-hop test might also reflect a “fast and controlled” ankle spring that limits late-swing wobble and tiny errors in the swinging leg's foot orientation during the last ∼100–200 ms before ball contact—precisely the window in which ball placement is determined. In these professional players, being both strong enough to control the body motion and bouncy enough to control the supporting and swinging legs’ ankle reaction allowed them to maintain accuracy even at higher shot velocities. This finding adds nuance to the classic speed–accuracy trade-off concept (26–28, 61).

Generally, one might expect that attempting to move explosively (high reactive strength) would impair accuracy—as seen in some youth players where very explosive jumps were linked to poorer shot placement (27). However, our data is compatible with the idea that this trade-off can be mitigated at high skill levels. In athletes who possess adequate strength and technical coordination, greater reactive ability did not come at the cost of accuracy; indeed, it is correlated with better accuracy. One interpretation is that a player with a responsive, efficient support leg can more finely time their kick and adjust body positioning, reducing error in ball direction despite high velocities. His interpretation is consistent with evidence that support-leg balance, stability, and technical control are associated with better kicking or striking accuracy (24, 35, 62–64).

In our data, higher reactive strength did not appear to compromise accuracy, suggesting that explosive leg qualities may contribute to kicking precision when they are integrated with sufficient stability and strength. It also resonates with findings in other sports: for instance, a recent study in basketball found no detriment of lower-body strength on technical skill accuracy (65). Similar patterns are seen in martial arts kicking, where balance and technical control govern striking accuracy (62, 64). These observations emphasize that technique and strength must be integrated for optimal performance. In our results, players who combined higher IMTP force with higher RSI tended, on average, to produce more powerful shots on target.

From a training standpoint, this underscores the importance of developing a well-rounded power profile: simply increasing strength without reactive ability (or vice versa) may not yield optimal accuracy benefits. In contrast, training that enhances both maximal force and rapid elastic recoil (e.g., heavy strength training coupled with plyometrics) could improve a player's capacity to strike the ball both hard and precisely. We also note that our modeling approach found relative IMTP force to be a significant factor for the proficiency score, underscoring the value of power-to-weight ratio for overall shooting performance. This is a reminder that excessive mass without proportional strength gains might hinder the fine motor execution of explosive, coordinated, skilled movements (24, 45, 58, 66).

### 4.4 Non-significant metrics and specificity

While the key predictors discussed above emerged clearly, it is equally instructive to consider what did not significantly predict performance in our analysis. Notably, several variables often thought to influence kicking were excluded by the regression models, highlighting the importance of task-specific assessment (36, 37, 46, 68). For instance, drop jump metrics—such as jump height, contact time, or stiffness from a standard drop jump test—did not enter any of our final models for shot velocity or accuracy. One reason may be that a single maximal drop jump (which involves a large vertical rebound off two legs) is biomechanically quite different from the soccer shot movement, where plant-leg mechanics and swing timing dominate the outcome (9). A maximal DJ is a whole-body SSC task in which athletes freely use hip and knee excursion and technique to manipulate ground-contact strategy—reactivity is certainly involved, but the outcome is diffuse and highly technique-sensitive (36, 46).

By contrast, our accuracy/composite models favored the 10/5 repeated-hop peak RSI, a deliberately ankle-dominant assessment (minimal hip/knee bend) that better mirrors the short contacts preceding ball-foot impact (9, 37, 46). Higher 10/5 RSI likely indexes a “fast and controlled” ankle spring that limits late-swing wobble and tiny errors in the swinging foot orientation during the last ∼100–200 ms before ball contact—precisely the window in which small support-leg perturbations or swinging-leg ankle floppiness can propagate into ball placement. This interpretation aligns with evidence that lower-limb balance, control, and stiffness characteristics relate to placement accuracy (23–25, 37, 38). In short, when the outcome is standardized shot accuracy (rather than maximal jump rebound height), repeated low-amplitude hops appear to capture the ankle-specific control that translates into kicking precision better than DJ-based reactivity does, at least for this cohort.

Similarly, rate-of-force-development (RFD) measures from the IMTP (e.g., impulse at 100 ms or 250 ms) were not retained in the forward-entry models once peak force was accounted for. This does not mean explosive force is unimportant, but in our data, it showed high collinearity and redundancy with peak force—consistent with literature showing strong associations of IMTP peak force with athletic performance, while time-specific force metrics often covary with maximal strength capacity (31, 32, 47, 67). In practical terms, an athlete's maximal strength in the IMTP encapsulated most of the variance that early-phase RFD might also explain, so adding RFD metrics did not improve predictive power in the model. This is an interesting result, as it suggests that for well-trained players, focusing on improving maximal force output (and reactive efficiency) may cover the benefits that one might otherwise expect from enhancing “pure” RFD. It also implies there might be a ceiling or threshold effect: once an athlete can exert a large force, the ability to ramp up to that force extremely quickly could be less of a differentiator in a task like shooting, which has a longer movement duration than a 100–250 ms window (9).

Another subtle result was that CMJ time-to-takeoff (an explosiveness metric) showed a non-significant association with shot velocity (*p* = .10) that was directionally positive, but its effect appeared secondary once braking-phase control (braking impulse) was accounted for. Players who jumped off the ground more quickly tended to shoot slightly faster, which is intuitively consistent with explosive leg drive, but the effect was not strong enough to reach significance—likely overshadowed by the greater relevance of braking-phase control (e.g., braking impulse) for setting up an effective plant. This interpretation is in keeping with CMJ force–time studies highlighting the role of eccentric/braking characteristics in explosive tasks (18, 19).

Overall, by using a rigorous variable-selection approach, we clarified that certain commonly referenced metrics (drop jump performance, IMTP RFD, jump height, etc.) did not add unique value beyond the key predictors already discussed for this cohort. This finding may help refine training focus: for instance, a coach may not need to place primary emphasis on improving whole-body vertical stiffness (as measured by drop-jump RSI) if the immediate goal is to enhance shooting performance, whereas improving ankle-dominant stiffness (as indexed by repeated-hop RSI) appears more directly aligned with our observed associations in this cohort. It also underscores the need for specificity—tests that closely mirror the kinetic and kinematic demands of the kick provided more insight than general explosive tests. Practitioners should keep this point in mind when profiling and training athletes for kicking performance.

### 4.5 Limitations and future directions

Despite the clear patterns observed, this study has several limitations that should temper our conclusions. First, the sample consisted of a single team of professional indoor (arena) soccer players, which limits generalizability. The relatively small sample size (*n* = 32) also limits the precision of regression estimates and increases the risk of sample-specific patterns. These athletes likely share similar training backgrounds and competitive environments, and the indoor game (with its smaller field and faster pace) might place somewhat different emphasis on shooting skills than the traditional outdoor game. Caution is warranted in extending these findings to other populations—for example, youth players or elite outdoor soccer players—without further study. The relationships between neuromuscular metrics and performance could shift with age, skill level, or style of play (indeed, we noted earlier that some factors important in our cohort, like reactive strength, have been detrimental in less-trained groups). Secondly, we examined only dominant-foot shots under a standardized, low-pressure setting. Real match shooting involves additional variables (e.g., defensive pressure, fatigue, time constraints, decision-making) that we did not capture, and non-dominant foot performance might rely on somewhat different mechanics or strength balance, which we did not explore in this study. Third, our regression models—while significant—still left a substantial portion of variance unexplained, indicating that factors beyond the force-plate metrics (such as technical skill, approach angle, trunk and hip biomechanics, or psychological factors) play a large role in shooting success. We also did not include any direct measures of kicking technique or foot/leg kinematics in our analysis; integrating those with neuromuscular data could further illuminate how physical capacity translates to ball-flight outcomes. In terms of analysis, while our modeling strategy intentionally minimized redundancy and constrained predictor count, the models remain sample-specific. No independent holdout sample (or cross-validation) was available to verify model generalizability, and therefore the explained variance may be somewhat inflated. Reporting adjusted *R*^2^ partially addresses concerns about model complexity, but replication in independent samples is needed. Future studies with larger and more diverse samples should verify the robustness of these predictors—for instance, confirming that braking impulse remains a top factor in other teams, or that strength/reactive rebound metrics consistently relate to accuracy across various competitive levels. It would also be beneficial to investigate causal relationships through training interventions. For example, does specifically training to increase CMJ braking impulse (via eccentric overload or plyometric “braking” drills) lead to measurable improvements in shot speed? Similarly, is there a point of diminishing returns for strength's impact on accuracy—i.e., once a player can produce X Newtons in an IMTP relative to body weight, does additional strength no longer improve accuracy? Longitudinal training studies that manipulate strength and reactive ability could answer such questions and help establish thresholds for “how much is enough” in the context of shooting performance. Finally, exploring neuromuscular predictors under conditions of fatigue or pressure (more game-like scenarios) could add ecological validity.

Despite these limitations, our findings provide novel insight by linking a comprehensive force-plate test battery with actual ball-striking outcomes in professional players. In doing so, we identified outcome-specific neuromuscular profiles—an eccentric braking-driven profile for shot speed, and a strength-plus-reactivity profile for shot accuracy—that refine the understanding of soccer kicking performance. These results confirm the longstanding notion that both power and precision are vital for successful shooting, while offering a more nuanced explanation of how each is achieved. Future research and applied training can build on this knowledge to help players develop the targeted physical qualities that underpin faster, more accurate shots, ultimately enhancing their effectiveness on the field.

## Data Availability

The raw data supporting the conclusions of this article will be made available by the authors, without undue reservation.
