# Supplementary material for: Lower-body strength and power profiles and their relationships with shot velocity and accuracy in elite arena soccer players
Source: Front Sports Act Living. 2026 Mar 17;8:1766063. doi: 10.3389/fspor.2026.1766063 (PMC13036158; doi:10.3389/fspor.2026.1766063)
Supplement: Supplementary file 1 [file Table1.pdf]

TABLE S1

Forward-Entry Model Steps and Changes in Explained Variance (ΔR^2)

Outcome	Step	Predictor Entered	R^2	Adj R^2	ΔR^2	F-change (df1,df2)	p-change
**Velocity**	1	CMJ Braking Impulse	0.151	0.123	0.151	5.34 (1,30)	0.028
**Accuracy**	1	IMTP Peak Force	0.168	0.140	0.168	6.05 (1,30)	0.020
	2	+ 10/5 Peak RSI	0.362	0.318	0.194	8.80 (1,29)	0.006
**Composite**	1	IMTP Rel. Peak Force	0.182	0.155	0.182	6.69 (1,30)	0.015
	2	+ 10/5 Peak RSI	0.308	0.260	0.126	5.28 (1,29)	0.029

Removed prior to regression due to redundancy within constructs:

- ***Strength / Stability:*** *IMTP impulse 0–250 ms, IMTP RFD 0–250 ms (retained representative: IMTP peak force, IMTP rel. peak force)*
- ***Eccentric braking capacity:*** *CMJ eccentric peak force, CMJ eccentric rate of deceleration, CMJ stiffness (retained representative: CMJ eccentric braking impulse)*
- ***Propulsive explosive power:*** *CMJ concentric impulse, CMJ concentric peak force, CMJ RSI-mod (retained representative: CMJ jump height)*
- ***Loaded reactive strength:*** *DJ RSI, DJ jump height, DJ contact time, DJ stiffness (retained representative: DJ time-to-peak braking)*
- ***Ankle-Dominant Reactive Strength:*** *10/5 average RSI, contact time, flight time, stiffness (retained representative: 10/5 peak RSI)*

Evaluated in regression but did not meet entry criterion ($p > 0.05$):

- ***Propulsive explosive power:*** *CMJ jump height*
- ***Loaded reactive strength:*** *DJ time-to-peak braking force*

CMJ = Countermovement Maximal Jump; DJ = Drop Jump; RSI = Reactive Strength Index; RFD = Rate of Force Development; IMTP = Isometric Mid-Thigh Pull
